# Improving Arterial Blood Gas Sampling Practice in the Emergency Department: A Two-Cycle Clinical Audit

**DOI:** 10.7759/cureus.116425

**Published:** 2026-09-17

**Authors:** Waleed Khalid Khalafallah Khalid, Hamid Zafar, Aariba Bisati, Aiman Nazir, Leke Popoola, Abdur Faisal, Tamkeen Showkat, Azan Malik, Tarik Moussaoui

**Affiliations:** 1 Emergency Department, Queen Elizabeth Hospital, London, GBR

**Keywords:** abg (arterial blood gas), chronic obstructive pulmonary disease (copd), clinical audit cycles, emergency medicine procedures, local infiltrative anaesthesia, procedural pain, quality improvement projects, venous blood gas (vbg)

## Abstract

Background

Arterial blood gas (ABG) sampling is commonly performed in emergency care but is invasive and painful, particularly when repeated sampling is required. In selected patients with acute respiratory disease, including chronic obstructive pulmonary disease (COPD) exacerbations, venous blood gas (VBG) analysis can provide useful screening information and may avoid unnecessary arterial puncture. A venous PCO₂ below 6.0 kPa, when interpreted alongside satisfactory pulse oximetry and the clinical context, can help exclude significant arterial hypercapnia, while venous pH is generally sufficiently close to arterial pH for initial assessment. Despite this, clinicians may still default to arterial sampling, and measures intended to reduce procedural pain, such as local anaesthesia, are not always routinely used. We evaluated whether a targeted educational intervention could improve clinicians’ knowledge and reported practice relating to ABG sampling.

Methods

We conducted a two-cycle clinical audit in a UK emergency department. Cycle 1 included 59 clinicians and identified baseline gaps in VBG knowledge, local anaesthetic use and patient-centred sampling practice. A multimodal intervention was subsequently introduced, combining departmental teaching, a short educational video, visual prompts and reinforcement during clinical work. Cycle 2 included 30 clinicians. Prespecified measures assessed knowledge of when VBG may provide adequate initial information, routine skin antisepsis and local anaesthetic use. Ultrasound use for difficult arterial access and enquiry about patient pain or experience were also assessed. As responses were anonymous and could not be paired between cycles, the two samples were analysed independently.

Results

Mean VBG knowledge increased from 51.7% (n/N = 61/118) in Cycle 1 to 75.0% (n/N = 45/60) in Cycle 2, an absolute improvement of 23.3 percentage points (95% CI 7.2-39.4; p = 0.005; Hedges g = 0.61). The proportion of clinicians answering both VBG knowledge items correctly increased from 32.2% (n/N = 19/59) to 60.0% (n/N = 18/30). Recognition that a normal venous pH and PCO₂ does not automatically require arterial confirmation increased from 67.8% (n/N = 40/59) to 90.0% (n/N = 27/30), while recognition of the potential role of VBG with pulse oximetry increased from 35.6% (n/N = 21/59) to 60.0% (n/N = 18/30).

Local anaesthetic use increased from 6.8% (n/N = 4/59) to 23.3% (n/N = 7/30) (absolute difference 16.6 percentage points; 95% CI 1.7-34.6; p = 0.039), although it remained the largest gap from the prespecified standard. Routine skin antisepsis remained consistently high at 96.6% (n/N = 57/59) versus 96.7% (n/N = 29/30). Enquiry about patient pain or experience increased from 50.8% (n/N = 30/59) to 80.0% (n/N = 24/30) (difference 29.2 percentage points; 95% CI 7.9-45.4; p = 0.011). Ultrasound use for difficult arterial access increased from 47.5% (n/N = 28/59) to 66.7% (n/N = 20/30).

Conclusions

After the multimodal intervention, VBG knowledge and several aspects of reported ABG practice improved. Local anaesthetic use remained low despite some improvement, suggesting that education alone may not be enough to change this procedural behaviour. Appropriate use of VBG in selected patients, together with better analgesia when ABG sampling is required, may reduce unnecessary arterial puncture and procedural pain. Future cycles should directly measure ABG indications, procedural practice and patient-reported pain.

## Introduction

Arterial blood gas (ABG) analysis remains an important investigation in emergency medicine when accurate assessment of oxygenation, ventilation and acid-base status is required. However, arterial puncture is invasive and painful, and repeated sampling can add further discomfort for patients. Good ABG practice therefore requires not only appropriate sampling technique, but also careful consideration of whether an arterial sample is genuinely necessary and how procedural pain can be minimised when it is required [1,2].

Venous blood gas (VBG) analysis can provide useful screening information in selected patients with acute respiratory disease. Venous pH generally shows clinically useful agreement with arterial pH, while venous PCO₂ is more appropriately used as a screening measure than as a direct substitute for PaCO₂ [3-5]. In paired arterial and venous samples, a venous PCO₂ below 6.0 kPa (45 mmHg) has demonstrated high sensitivity for excluding significant arterial hypercapnia [3,4]. The British Thoracic Society also recognises that, in selected patients who are not at risk of metabolic acidosis, a satisfactory oxygen saturation on pulse oximetry combined with a venous PCO₂ below 6.0 kPa may provide sufficient initial screening and can help avoid unnecessary arterial puncture [4]. Prospective evidence in patients presenting with acute exacerbations of chronic obstructive pulmonary disease (COPD) has similarly shown that a VBG-based assessment combined with pulse oximetry could substantially reduce the number of patients requiring immediate arterial sampling [5].

This approach should not be interpreted as replacing ABG in all patients. Current National Institute for Health and Care Excellence (NICE) guidance recommends ABG measurement in patients presenting to hospital with an acute exacerbation of COPD [6]. Arterial sampling remains necessary when accurate arterial oxygenation is required, when patients are severely unwell or deteriorating, when venous results are abnormal or inconsistent with the clinical picture, or when management decisions such as ventilatory support depend on arterial measurements [4,6]. The practical issue is therefore not simply whether VBG or ABG is preferable, but whether clinicians can use VBG appropriately as a screening tool and recognise the circumstances in which arterial sampling adds clinically important information. Similarly, normal venous pH and PCO₂ do not automatically require confirmation with a painful ABG solely because the initial sample was venous; the decision should be guided by the clinical context, pulse oximetry and the specific information required [3-5].

When arterial sampling is required, minimising procedural pain should form part of good ABG practice. Appropriate skin antisepsis is recommended before arterial puncture [1]. Local anaesthetic infiltration, including lidocaine, is an effective method of reducing arterial puncture-related pain and is recommended unless clinical urgency precludes its use [2,4]. Evidence from a randomised double-blind placebo-controlled trial showed that arterial puncture was less painful when local anaesthesia was used, while infiltration itself was no more painful than placebo and did not make the procedure more difficult [7]. A subsequent systematic review and meta-analysis similarly found local anaesthetic infiltration to be among the most effective interventions for reducing arterial puncture-related pain and supported its routine use [2]. These findings directly challenge the misconception that lidocaine injection causes pain comparable to the arterial puncture itself and support its use when an ABG is required [7]. Ultrasound guidance may additionally facilitate radial arterial puncture in technically difficult cases and can be used selectively when conventional palpation is challenging [8].

The first audit cycle identified gaps in clinicians’ knowledge regarding the appropriate use of VBG and in reported practices intended to improve the safety and patient experience of arterial sampling, particularly the use of local anaesthesia. These findings provided the rationale for a targeted multimodal educational intervention.

The aim of this two-cycle clinical audit was to determine whether the intervention improved clinicians’ knowledge and reported ABG sampling practice compared with the pre-intervention baseline. The primary areas assessed were VBG knowledge, routine skin antisepsis and local anaesthetic use, with ultrasound use for difficult arterial access and enquiry about patient pain or experience evaluated as secondary measures.

## Materials and methods

Study design and setting

This was a prospective two-cycle clinical audit conducted in the Emergency Department at Queen Elizabeth Hospital, Woolwich, Lewisham and Greenwich NHS Trust, London, UK. The project evaluated clinicians’ knowledge and reported practice relating to ABG sampling before and after a targeted educational intervention.

Cycle 1 served as the pre-intervention baseline and included 59 clinician responses. Following implementation of the intervention, the same retained core measures were reassessed in Cycle 2. The second-cycle questionnaire was open from 27 July to 14 August 2026.

The project was reported in accordance with the Standards for Quality Improvement Reporting Excellence (SQUIRE 2.0) [9].

Participants and data collection

Clinicians involved in ABG sampling in the Emergency Department were invited to complete an anonymous electronic questionnaire. Thirty Cycle 2 responses documented consent and were included in the analysis. The retained audit outcome fields were complete.

Because responses were anonymous, individual participants could not be linked between Cycle 1 and Cycle 2. The two cycles were therefore analysed as independent cross-sectional samples rather than paired observations.

The questionnaire recorded clinical grade, two VBG knowledge items, routine skin antisepsis, local anaesthetic use, ultrasound use for radial arterial access and enquiry about patient pain or experience following ABG sampling. A separate Cycle 2 question assessed reported exposure to the educational video as a process measure. The staff questionnaire is reproduced in the Appendices, with all questionnaire items and response options presented in tabular form; the Cycle 2-only educational video item is identified separately as a process measure.

Audit standards and outcome measures

Three prespecified primary audit standards were retained across both cycles. The first was VBG knowledge, defined as the mean percentage score across two knowledge items, with a target of at least 80%. The second was skin antisepsis, defined as reported routine cleansing of the skin with alcohol or chlorhexidine before arterial puncture, with a target of 100%. The third was local anaesthetic use, defined as reported use of local anaesthesia before arterial puncture, with a prespecified interim target of at least 70%.

Two additional measures were analysed as secondary exploratory outcomes. Ultrasound use was considered aligned when respondents reported using ultrasound either routinely or in difficult arterial access. Patient-experience enquiry was considered aligned when respondents reported asking the patient about pain or their experience following ABG sampling.

The two VBG knowledge items were based on published screening approaches described by Kelly et al., the British Thoracic Society, and McKeever et al. [3-5]. The skin antisepsis standard was based on World Health Organization guidance [1]. The local anaesthetic standard was informed by British Thoracic Society guidance and evidence showing reduced arterial puncture-related pain [2,4,7]. The 70% local anaesthetic threshold was an interim departmental improvement target rather than a diagnostic criterion.

The first VBG knowledge item assessed recognition that VBG interpreted alongside pulse oximetry may provide sufficient initial information in selected clinical circumstances. The second assessed recognition that normal venous pH and PCO₂ do not automatically require arterial confirmation solely because the initial specimen was venous. The two items were prespecified as complementary components of a single VBG knowledge domain rather than separate primary audit standards.

Educational intervention

Following Cycle 1, a multimodal educational intervention was implemented rather than relying on a single teaching encounter. The intervention comprised a departmental teaching presentation, a recorded educational video, a visual poster displayed within the department and repeated reinforcement of the key messages during routine clinical work.

The educational content addressed five areas identified within the audit framework: appropriate interpretation of VBG alongside pulse oximetry; recognition of circumstances in which ABG remained necessary; routine skin antisepsis; use of local anaesthesia to reduce arterial puncture-related pain; and selective ultrasound use for difficult radial arterial access. Patient comfort and enquiry about procedural pain or experience were also emphasised.

The educational intervention was intended to influence both clinical knowledge and procedural practice. Reported viewing of the educational video was recorded in Cycle 2 as a measure of intervention reach but was not treated as evidence of attention, knowledge retention or a causal relationship between exposure and subsequent responses.

Questionnaire definitions and scoring

Responses were classified using prespecified definitions applied consistently to the retained audit framework.

For each of the two VBG knowledge items, the response aligned with the audit definition was assigned a score of 1 and all other responses a score of 0. A participant-level VBG knowledge score was calculated as:

$\text{VBG knowledge score} = \frac{\text{VBG item 1 score} + \text{VBG item 2 score}}{2}$

Possible individual scores were therefore 0%, 50% or 100%. The cycle-level VBG knowledge outcome was the mean participant score.

For skin antisepsis, only the response indicating routine use of alcohol or chlorhexidine was classified as aligned. For local anaesthesia, reported use before arterial puncture was classified as aligned. For the exploratory ultrasound measure, both routine ultrasound use and use in difficult cases were classified as aligned. Patient-experience enquiry was classified as aligned when the respondent reported asking the patient about pain or their experience after sampling.

Statistical analysis

Data were analysed using Microsoft Excel for Microsoft 365 (Microsoft Corporation, Redmond, WA).

The mean VBG knowledge score was reported with standard deviation and a t-based 95% confidence interval. Percentage outcomes were reported with the corresponding numerator and denominator in the format % (n/N = x/y), together with 95% confidence intervals where applicable. For each of the three primary standards, performance was also expressed as the absolute percentage-point difference from the prespecified target.

Because the questionnaires were anonymous and participants could not be linked across cycles, Cycle 1 and Cycle 2 were treated as independent samples. The difference in mean VBG knowledge score between cycles was assessed using Welch’s t-test, and Hedges g was calculated as a standardised effect estimate.

For binary outcomes, changes were expressed as the Cycle 2 percentage minus the Cycle 1 percentage. Newcombe-Wilson 95% confidence intervals were calculated for absolute percentage-point differences, and two-sided Fisher’s exact tests were used for exploratory comparisons between cycles.

Wilson confidence intervals and Newcombe-Wilson intervals for differences between independent proportions were calculated using established score-based methods [10,11]. Hedges g was calculated with the small-sample bias correction described by Hedges [12].

All statistical tests were two-sided. p-values were considered exploratory and were not adjusted for multiple comparisons. Findings were therefore interpreted in conjunction with effect estimates, 95% confidence intervals, prespecified target attainment, clinical relevance and the limitations of the uncontrolled two-cycle audit design.

## Results

Study sample and overall findings

Cycle 1 included 59 clinician responses and established the pre-intervention baseline, while Cycle 2 included 30 clinician responses following the educational intervention. Because responses were anonymous, participants could not be matched between cycles and the two datasets were analysed as independent samples.

The clinical-grade distribution was broadly similar between cycles (Table 1).

**Table 1:** Clinical-grade distribution of respondents in Cycle 1 and Cycle 2. Values are n (%), with percentages calculated using the total number of respondents in each cycle. The Cycle 1 GP ST1 response was grouped as GP trainee, and the two ACP response variants were combined. ACP, advanced clinical practitioner; F2, Foundation Year 2; GP, general practitioner; JCF, junior clinical fellow; SCF, senior clinical fellow; SHO, senior house officer

Clinical grade	Cycle 1 (n = 59)	Cycle 2 (n = 30)
F2	11 (18.6%)	6 (20.0%)
JCF/SHO	31 (52.5%)	15 (50.0%)
SCF/Registrar	14 (23.7%)	8 (26.7%)
ACP	2 (3.4%)	0 (0.0%)
GP trainee	1 (1.7%)	1 (3.3%)
Total	59 (100.0%)	30 (100.0%)

Across the retained audit framework, Cycle 2 showed improvement in most measured domains. The mean VBG knowledge score increased from 51.7% (n/N = 61/118) in Cycle 1 to 75.0% (n/N = 45/60) in Cycle 2, local anaesthetic use increased from 6.8% (n/N = 4/59) to 23.3% (n/N = 7/30) and patient-experience enquiry increased from 50.8% (n/N = 30/59) to 80.0% (n/N = 24/30). Routine skin antisepsis remained consistently high at 96.6% (n/N = 57/59) in Cycle 1 and 96.7% (n/N = 29/30) in Cycle 2. Ultrasound use for difficult arterial access increased from 47.5% (n/N = 28/59) to 66.7% (n/N = 20/30).

The complete comparison between cycles is presented in Table 2.

**Table 2:** Comparison of Cycle 1 and Cycle 2 outcomes across the retained audit framework. Positive changes favour Cycle 2. VBG item 1 assessed recognition that VBG interpreted alongside pulse oximetry may provide sufficient initial information in selected circumstances. VBG item 2 assessed recognition that normal venous pH and PCO₂ do not automatically require arterial confirmation. Comparisons were exploratory because the two cycles consisted of independent samples and multiple outcomes were assessed without adjustment for multiplicity. Welch’s t-test was used for the mean VBG knowledge comparison, and two-sided Fisher’s exact tests were used for binary outcomes. Fisher’s exact test does not generate a t, χ² or F statistic, so the corresponding test-statistic cells are reported as N/A. For the mean VBG knowledge row, n/N represents aligned knowledge-item responses across the two retained items; Welch’s t-test was performed on participant-level mean scores. CI, confidence interval; VBG, venous blood gas; PCO₂, partial pressure of carbon dioxide; pp, percentage points

Outcome	Cycle 1	Cycle 2	Change (pp)	95% CI	p	Test	Statistic
Mean two-item VBG knowledge score	51.7% (n/N = 61/118)	75.0% (n/N = 45/60)	23.3	7.2 to 39.4	0.005	Welch’s t-test	t = 2.89
Routine skin antisepsis	96.6% (n/N = 57/59)	96.7% (n/N = 29/30)	0.1	-13.5 to 8.7	1	Fisher’s exact test	N/A
Local anaesthetic use	6.8% (n/N = 4/59)	23.3% (n/N = 7/30)	16.6	1.7 to 34.6	0.039	Fisher’s exact test	N/A
VBG knowledge item 1	35.6% (n/N = 21/59)	60.0% (n/N = 18/30)	24.4	2.6 to 43.3	0.042	Fisher’s exact test	N/A
VBG knowledge item 2	67.8% (n/N = 40/59)	90.0% (n/N = 27/30)	22.2	3.4 to 36.5	0.036	Fisher’s exact test	N/A
Ultrasound use	47.5% (n/N = 28/59)	66.7% (n/N = 20/30)	19.2	-2.6 to 37.9	0.116	Fisher’s exact test	N/A
Patient-experience enquiry	50.8% (n/N = 30/59)	80.0% (n/N = 24/30)	29.2	7.9 to 45.4	0.011	Fisher’s exact test	N/A

Primary audit standards

None of the three prespecified primary standards was fully achieved in Cycle 2, although the degree of residual non-attainment differed markedly between measures. Mean VBG knowledge reached 75.0% (n/N = 45/60), five percentage points below the ≥80% target. Routine skin antisepsis reached 96.7% (n/N = 29/30), only 3.3 percentage points below the strict 100% standard. Local anaesthetic use reached 23.3% (n/N = 7/30) and remained 46.7 percentage points below the interim target of ≥70%, representing the largest remaining gap. Cycle 2 performance against these three prespecified standards is shown in Figure 1.

**Figure 1:**
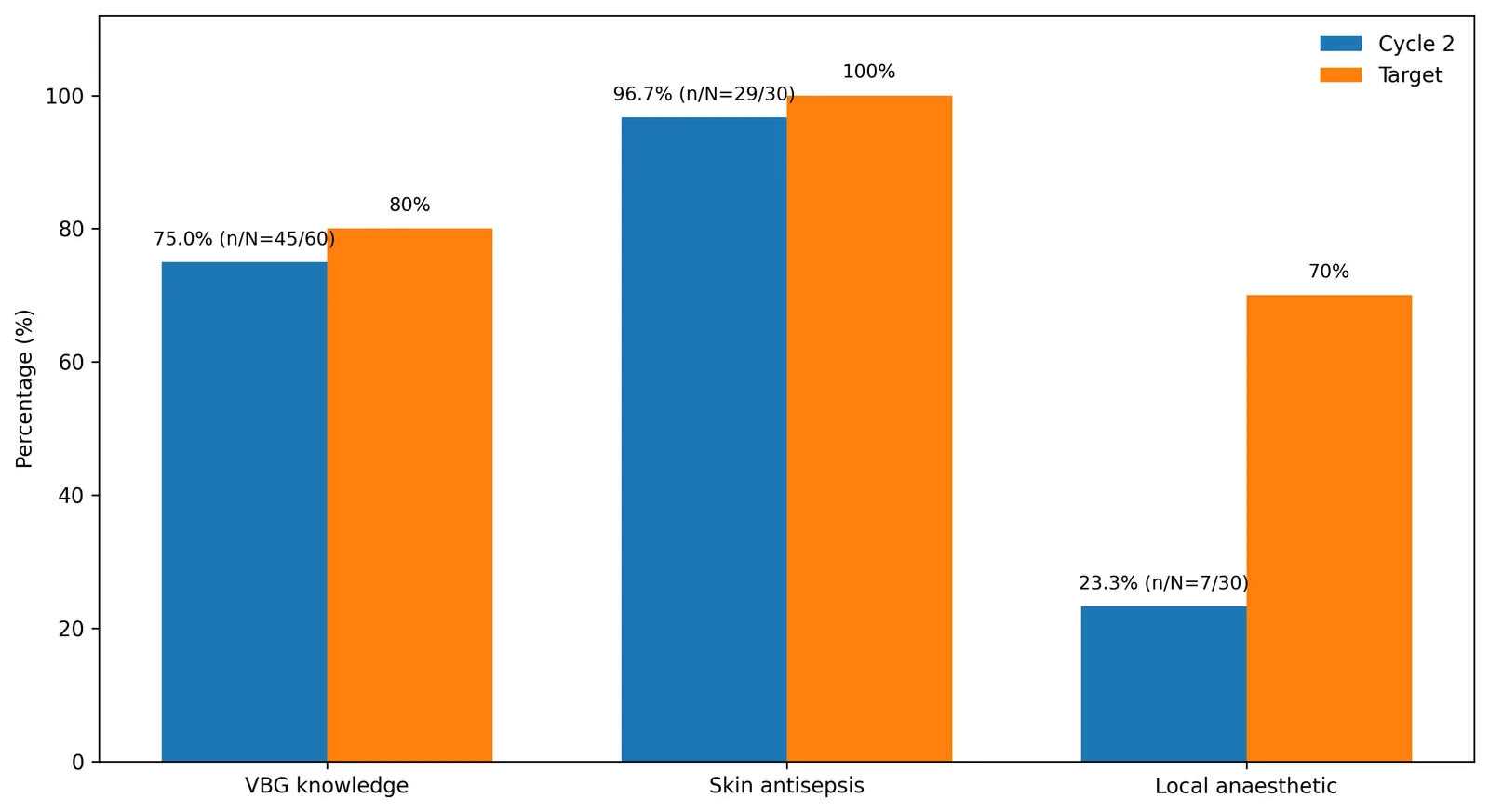
Cycle 2 performance against the three prespecified primary audit standards. Cycle 2 performance is shown alongside the prespecified target for each primary audit domain. VBG knowledge represents the mean percentage score across the two retained knowledge items. Skin antisepsis represents reported routine cleansing with alcohol or chlorhexidine before arterial puncture. Local anaesthetic represents reported use before arterial puncture. Cycle 2 values were mean VBG knowledge 75.0% (n/N = 45/60), skin antisepsis 96.7% (n/N = 29/30) and local anaesthetic use 23.3% (n/N = 7/30). For VBG knowledge, n/N = 45/60 denotes 45 aligned responses across the two knowledge items out of 60 evaluable item responses from 30 clinicians. VBG, venous blood gas

VBG knowledge

The mean two-item VBG knowledge score rose from 51.7% (n/N = 61/118) in Cycle 1 to 75.0% (n/N = 45/60) in Cycle 2 (Cycle 2 SD 34.1%; 95% CI 62.3%-87.7%). The absolute improvement was 23.3 percentage points (95% CI 7.2-39.4; Welch’s t = 2.89, df = 66.34; p = 0.005; Hedges g = 0.61). The 80% target was not reached, but Cycle 2 was closer to the standard.

The distribution of participant scores also shifted towards complete alignment (Figure 2). In Cycle 1, 28.8% (n/N = 17/59) scored 0%, 39.0% (n/N = 23/59) scored 50% and 32.2% (n/N = 19/59) achieved 100% across the two VBG items. In Cycle 2, 10.0% (n/N = 3/30) scored 0%, 30.0% (n/N = 9/30) scored 50% and 60.0% (n/N = 18/30) achieved full alignment.

**Figure 2:**
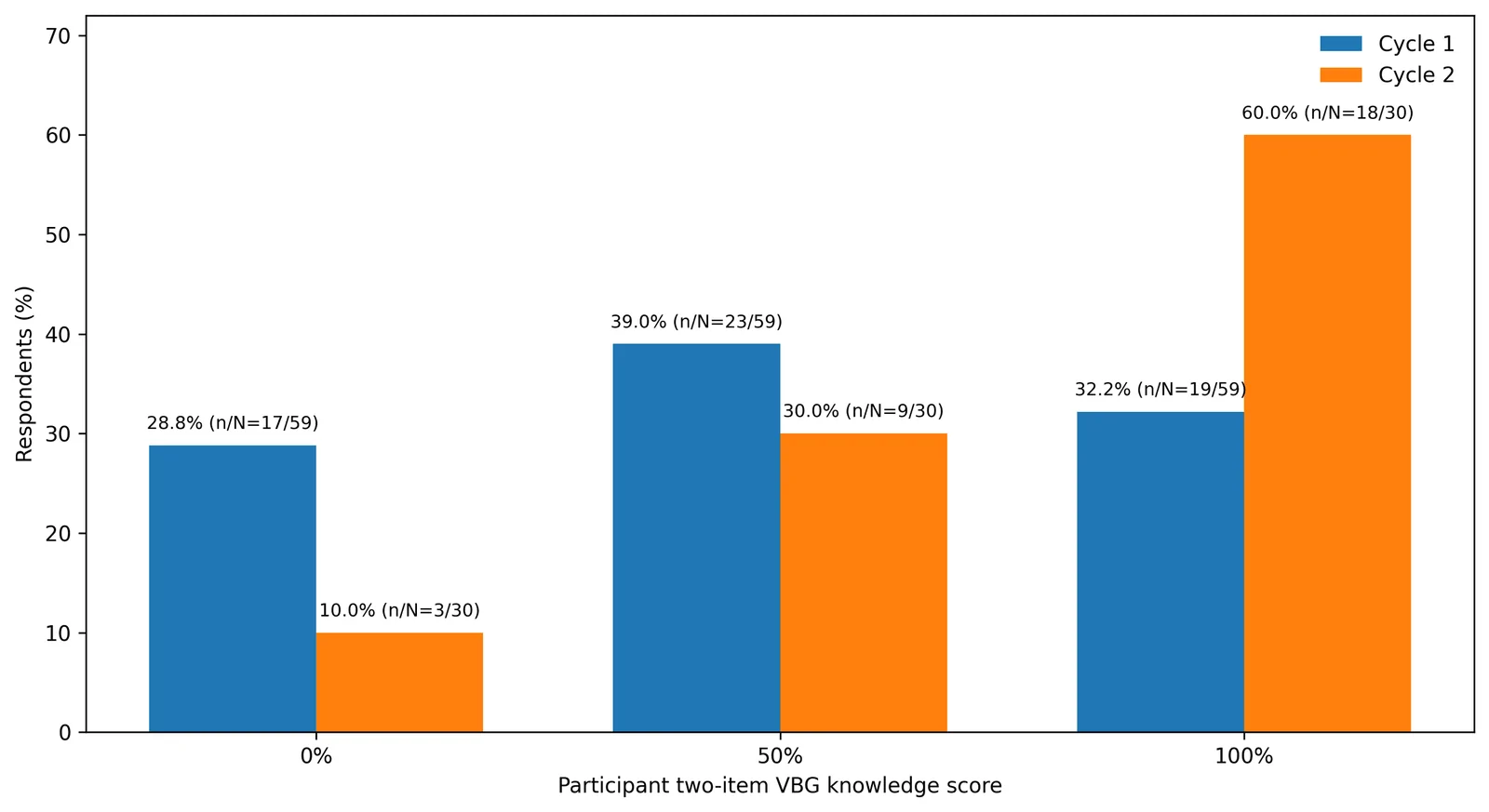
Distribution of participant VBG knowledge scores in Cycle 1 and Cycle 2. Participant scores were based on the two retained VBG knowledge items, giving possible scores of 0%, 50% or 100%. The proportion achieving full alignment increased from 32.2% (n/N = 19/59) in Cycle 1 to 60.0% (n/N = 18/30) in Cycle 2, while the proportion scoring 0% decreased from 28.8% (n/N = 17/59) to 10.0% (n/N = 3/30). In Cycle 1, the 0%, 50% and 100% score groups were 28.8% (n/N = 17/59), 39.0% (n/N = 23/59) and 32.2% (n/N = 19/59), respectively; in Cycle 2 they were 10.0% (n/N = 3/30), 30.0% (n/N = 9/30) and 60.0% (n/N = 18/30). VBG, venous blood gas

Improvement was seen in both components of the VBG knowledge domain. Recognition that VBG interpreted alongside pulse oximetry may provide sufficient initial information in selected circumstances increased from 35.6% (n/N = 21/59) to 60.0% (n/N = 18/30) (difference +24.4 percentage points; 95% CI 2.6-43.3; two-sided Fisher’s exact p = 0.042). Recognition that normal venous pH and PCO₂ do not automatically require arterial confirmation increased from 67.8% (n/N = 40/59) to 90.0% (n/N = 27/30) (difference +22.2 percentage points; 95% CI 3.4-36.5; two-sided Fisher’s exact p = 0.036).

The two knowledge items were prespecified components of a single VBG knowledge domain and were therefore interpreted descriptively rather than as separate primary audit standards.

Skin antisepsis and local anaesthetic use

Routine skin cleansing with alcohol or chlorhexidine remained consistently high across both cycles. It was reported by 96.6% (n/N = 57/59) of respondents in Cycle 1 and 96.7% (n/N = 29/30; 95% CI 83.3%-99.4%) in Cycle 2. The absolute change was +0.1 percentage points (95% CI -13.5 to 8.7; two-sided Fisher’s exact p = 1.000). The strict 100% standard was therefore missed by one Cycle 2 respondent.

A more marked change was observed in local anaesthetic use. Reported use before arterial puncture increased from 6.8% (n/N = 4/59) of respondents in Cycle 1 to 23.3% (n/N = 7/30; 95% CI 11.8%-40.9%) in Cycle 2. This represented an absolute increase of 16.6 percentage points (95% CI 1.7-34.6; two-sided Fisher’s exact p = 0.039). Despite this improvement, local anaesthetic use remained substantially below the prespecified interim target of ≥70%.

Among the 23 Cycle 2 responses not meeting the local anaesthetic definition, 18 reported not using local anaesthesia, two selected the response indicating that lidocaine injection itself was painful and three described conditional or occasional use.

Secondary exploratory outcomes

Ultrasound use either routinely or for difficult arterial access increased from 47.5% (n/N = 28/59) in Cycle 1 to 66.7% (n/N = 20/30; 95% CI 48.8%-80.8%) in Cycle 2. The absolute increase was 19.2 percentage points (95% CI -2.6 to 37.9; two-sided Fisher’s exact p = 0.116). All 20 aligned Cycle 2 responses described ultrasound use in difficult cases; none reported routine use.

Patient-experience enquiry showed the largest absolute improvement among the binary measures, increasing from 50.8% (n/N = 30/59) in Cycle 1 to 80.0% (n/N = 24/30; 95% CI 62.7%-90.5%) in Cycle 2. The absolute increase was 29.2 percentage points (95% CI 7.9-45.4; two-sided Fisher’s exact p = 0.011).

This measure reflected clinician-reported enquiry about pain or experience after ABG sampling and did not directly measure patient-reported pain, comfort or satisfaction.

## Discussion

Principal findings

Following the educational intervention, VBG knowledge improved, and clinicians reported better practice in several areas. Mean VBG knowledge increased from 51.7% (n/N = 61/118) in Cycle 1 to 75.0% (n/N = 45/60) in Cycle 2, an improvement of 23.3 percentage points with a moderate effect size (Hedges g = 0.61). Local anaesthetic use rose from 6.8% (n/N = 4/59) to 23.3% (n/N = 7/30), while patient-experience enquiry increased from 50.8% (n/N = 30/59) to 80.0% (n/N = 24/30). Skin antisepsis remained high at 96.6% (n/N = 57/59) and 96.7% (n/N = 29/30) in the two cycles.

Improvement was seen across more than one outcome. Both VBG knowledge items improved, more participants achieved a full VBG knowledge score and several reported practice measures also improved. These findings suggest that the changes after the intervention were not limited to a single questionnaire item.

None of the three primary standards was fully achieved in Cycle 2. However, the remaining gaps differed considerably. VBG knowledge was close to the 80% target, skin antisepsis remained close to 100%, while local anaesthetic use remained well below the 70% interim target. These results help identify which areas improved after the first intervention and which require further work.

VBG knowledge and selection of arterial sampling

The improvement in VBG knowledge is clinically important because arterial sampling should be performed when arterial measurements are required rather than as an automatic default. Venous pH has clinically useful agreement with arterial pH in selected acute respiratory presentations, while venous PCO₂ can be used as a screening measure for significant arterial hypercapnia [3-5]. A venous PCO₂ below 6.0 kPa has demonstrated high sensitivity for excluding significant arterial hypercapnia, and the British Thoracic Society recognises a role for VBG interpreted alongside pulse oximetry in appropriately selected patients [3,4].

A recent systematic review of adults with respiratory presentations also highlighted that venous measurements are not interchangeable with arterial measurements for every variable. Venous pH may provide clinically useful information, whereas venous PCO₂ and particularly PO₂ are less suitable as direct substitutes for arterial values [13]. This supports the selective screening approach used in our audit rather than routine replacement of ABG with VBG.

The two VBG knowledge items showed different levels of alignment. By Cycle 2, 90.0% (n/N = 27/30) of respondents recognised that normal venous pH and PCO₂ do not automatically mandate arterial confirmation solely because the initial sample was venous. In contrast, 60.0% (n/N = 18/30) recognised that VBG interpreted alongside pulse oximetry may provide sufficient initial information in selected circumstances.

This may indicate that clinicians were more confident recognising when a reassuring VBG did not need arterial confirmation than deciding to use a venous-based assessment from the outset. The latter therefore remains an area for further education.

The aim is not to replace ABG with VBG in all patients. NICE continues to recommend ABG assessment for patients presenting to hospital with acute COPD exacerbations [6], and ABG remains necessary when accurate arterial oxygenation is required, illness is severe or worsening, findings do not fit the clinical picture or management decisions, such as ventilatory support depend on arterial measurements [4,6]. Arterial sampling should therefore be used selectively when arterial information is clinically required.

Previous quality-improvement work has shown that education and guidance can reduce unnecessary ABG testing. Martínez-Balzano et al. reported lower ABG utilisation and improved appropriateness of testing after an educational intervention across several intensive care units [14]. Our audit did not measure actual ABG ordering or indications, so it cannot show that unnecessary arterial puncture was reduced. Measuring ABG use and clinical indications directly would therefore be useful in the next cycle.

Local anaesthesia: the major implementation gap

The main remaining procedural gap was local anaesthetic use. Reported use increased from 6.8% (n/N = 4/59) to 23.3% (n/N = 7/30), but it remained well below the prespecified interim target.

This remains important because local anaesthesia can reduce pain during arterial puncture [2,7]. Lightowler and Elliott found that local anaesthesia reduced pain without making arterial puncture more difficult, and the discomfort from infiltration did not remove its overall analgesic benefit [7].

Low use of local anaesthesia is not unique to our department. In a UK survey of 178 doctors across eight hospitals, Sado and Deakin reported routine local anaesthetic use for ABG sampling in approximately 60% (n/N = 46/77) of anaesthetists, 2% (n/N = 1/50) of physicians and 0% (n/N = 0/51) of surgeons [15]. Although that study was conducted in a different setting and is older, it shows that underuse of local anaesthesia has been recognised for many years. Our findings suggest that this implementation gap may still persist in routine practice.

Two Cycle 2 respondents reported avoiding local anaesthesia because they believed the lidocaine injection itself was painful. This suggests that concern about the additional injection may still influence practice and should be addressed in future teaching.

Although local anaesthetic use increased from baseline, 76.7% (n/N = 23/30) of Cycle 2 respondents still did not meet the audit definition. Education alone may therefore not be enough to achieve routine use. Access to lidocaine, established procedural habits, time pressure and familiarity with administration may also affect practice, although these factors were not measured in this audit.

The next intervention should therefore address practical barriers as well as knowledge. Possible measures include making lidocaine readily available at the point of care, providing brief guidance on its use and including analgesia routinely in ABG procedural teaching.

Patient-centred and procedural practice

Patient-experience enquiry increased from 50.8% (n/N = 30/59) to 80.0% (n/N = 24/30), an absolute increase of 29.2 percentage points. This suggests greater attention to the patient’s experience of arterial sampling, but the audit measured whether clinicians reported asking about pain or experience; it did not directly measure the patient’s pain.

A future cycle should include a patient-reported pain score after arterial puncture. This would allow direct assessment of patient discomfort and whether greater use of local anaesthesia is associated with less pain.

Skin antisepsis was already high in Cycle 1 and remained high in Cycle 2, leaving little scope for further improvement.

Ultrasound use increased from 47.5% (n/N = 28/59) to 66.7% (n/N = 20/30), although the 95% confidence interval for the difference crossed zero and the exploratory Fisher’s exact comparison was not statistically significant (p = 0.116). All aligned Cycle 2 responses described ultrasound use in difficult cases rather than routinely, consistent with its intended selective use. Randomised evidence supports ultrasound guidance in difficult radial arterial puncture [8].

Strengths and limitations

The audit completed a full cycle of baseline measurement, intervention and re-audit. The same core measures were retained across both cycles, and the audit standards and outcome definitions were specified in advance. Assessing several related domains also provided a broader picture of reported ABG sampling practice.

We reported absolute differences and 95% confidence intervals in addition to percentages. Because individual respondents could not be matched between cycles, the two samples were analysed independently. A standardised effect size was also reported for the VBG knowledge outcome.

The study also has important limitations. Most outcomes reflected self-reported knowledge and practice rather than directly observed clinical behaviour and may therefore be affected by recall or social-desirability bias. The anonymous design prevented individual-level matching between cycles, and the smaller Cycle 2 sample resulted in wider confidence intervals for several estimates.

The two-item VBG knowledge measure was designed for this local audit rather than as a validated psychometric instrument and should therefore be interpreted as a pragmatic measure of alignment with the audit framework. The project was also conducted in a single Emergency Department, which may limit generalisability.

Most importantly, the audit did not directly measure ABG utilisation, clinical indication, first-pass success, procedural complications, actual administration of local anaesthetic during individual procedures or patient-reported pain. It cannot therefore demonstrate that fewer unnecessary ABGs were performed or that procedural pain was reduced.

Finally, the uncontrolled before-and-after design does not establish causation. Differences in the respondents between cycles, staff turnover, concurrent learning and other temporal factors may have contributed to the observed changes. Exploratory p-values were also not adjusted for multiple comparisons and should be interpreted alongside effect estimates, confidence intervals and clinical relevance. Although the clinical-grade distribution was broadly similar between cycles, differences in clinician experience may have influenced the findings, as analyses were not adjusted for grade.

Implications for practice and future improvement

The next audit cycle should focus more on practical changes in the department rather than education alone.

For VBG use, a simple decision aid could help clinicians decide when VBG with pulse oximetry may provide sufficient initial information and when an ABG is still required. For local anaesthesia, lidocaine should be easy to access, with brief practical guidance available at the point of care.

The next cycle should also incorporate direct observation of clinical practice. Prospective ABG assessment should record the indication for sampling, whether local anaesthetic was administered, the number of puncture attempts, ultrasound use where appropriate and procedural success. Direct patient-reported pain scores should be added, alongside measurement of actual ABG utilisation.

Keeping the existing core measures while adding direct clinical outcomes would allow comparison with earlier cycles and provide a better assessment of actual patient care.

The second cycle showed improvement in several areas but also identified local anaesthetic use as an important remaining gap. This should be a priority for the next intervention.

## Conclusions

This two-cycle clinical audit found improvements in VBG knowledge, patient-experience enquiry and reported local anaesthetic use after a multimodal educational intervention. Skin antisepsis remained high in both cycles. However, local anaesthetic use remained well below the prespecified target and was the main area requiring further improvement. The findings suggest that education can improve knowledge but may have less effect on established procedural practice. Further work should therefore combine education with practical measures, including easier access to local anaesthesia and clear point-of-care guidance. Future audit cycles should also measure ABG indications, actual procedural practice and patient-reported pain directly to determine whether changes in clinician knowledge lead to more selective arterial sampling and a better patient experience.

## Appendices

Staff questionnaire

Participant information. This questionnaire is part of a quality improvement (audit) project aimed at improving the care provided to patients undergoing arterial blood gas (ABG) sampling in the Emergency Department. The focus is on patient pain and comfort, infection prevention and control and procedural safety. Please answer the questions based on your actual daily practice, not the ideal or recommended practice. Responses are anonymous, and no personal identifiers are collected.

Note: The Cycle 2-only educational-video intervention-reach item is shown separately at the end of the table. It was recorded as a process measure and was not included in the retained audit outcome analysis. 

**Table 3:** Staff questionnaire used in the clinical audit. The questionnaire was used to assess clinicians’ knowledge and reported practice relating to arterial blood gas sampling. Items 1-8 formed the retained audit questionnaire used for outcome analysis. Item 9, assessing exposure to the educational video, was included in Cycle 2 only as a process measure and was not included in the retained audit outcome analysis. ABG, arterial blood gas; COPD, chronic obstructive pulmonary disease; F2, Foundation Year 2; JCF, junior clinical fellow; PCO₂, partial pressure of carbon dioxide; SCF, senior clinical fellow; SHO, senior house officer; VBG, venous blood gas

Item	Question	Response options
Section A: Participation and role		
1. Consent	I agree to participate in this anonymous clinical audit questionnaire.	Yes. No. If you select No, please do not complete the remaining questions.
2. Current clinical level	What is your current clinical level?	F2. JCF/SHO SCF/Registrar. ACP. GP trainee. Other (please specify): ______________________________
Section B: VBG knowledge		
3. Selected COPD exacerbations	In your opinion, can pulse oximetry combined with a VBG be sufficient in cases of COPD exacerbation?	Yes. No, we have to obtain an arterial sample (ABG). Other (please specify): ______________________________
4. Normal venous pH and PCO₂	If pH and PCO₂ are normal on a VBG sample, do we have to perform an ABG because venous samples are inaccurate?	Yes. No. Other (please specify): ______________________________
Section C: ABG sampling practice		
5. Skin antisepsis	In your daily practice, before taking an ABG sample, do you clean the skin using alcohol or chlorhexidine?	Yes, routinely. No, I often skip this step due to time or pressure. Other (please specify): ______________________________
6. Local anaesthetic	Do you use local anaesthesia (e.g. lidocaine) before arterial puncture?	Yes. No. No, because lidocaine injection itself is painful. Other (please specify): ______________________________
7. Ultrasound use	Do you use ultrasound to localise the radial artery?	Yes, routinely. Yes, in difficult cases. No, I rely on palpation. Other (please specify): ______________________________
8. Patient experience	After performing ABG sampling, do you ask the patient about their experience or level of pain?	Yes. No. Other (please specify): ______________________________
Cycle 2 intervention-reach item		
9. Educational video exposure (Cycle 2 only)	Have you watched the educational video?	I have watched the educational video. I have not watched the educational video.
